# Inducing Affective Learning Biases with Cognitive Training and Prefrontal tDCS: A Proof-of-Concept Study

**DOI:** 10.1007/s10608-020-10146-9

**Published:** 2020-10-05

**Authors:** Margot Juliëtte Overman, Michael Browning, Jacinta O’Shea

**Affiliations:** 1 Wellcome Centre for Integrative Neuroimaging, FMRIB, Nuffield Department of Clinical Neurosciences, University of Oxford, Oxford, OX3 9DU England; 2 Department of Psychiatry, University of Oxford, Warneford Hospital, Oxford, OX3 7JX England; 3 Oxford Health NHS Foundation Trust, Warneford Hospital, Warneford Lane, Oxford, OX3 7JX England; 4 Oxford Centre for Human Brain Activity (OHBA), Wellcome Centre for Integrative Neuroimaging, Department of Psychiatry, University of Oxford, Oxford, OX3 7JX England

**Keywords:** Depression, Anxiety, Computational psychiatry, tDCS, Transcranial direct current stimulation, Non-invasive brain stimulation, Affective disorders, Volatility, Reinforcement learning, Reward, dlPFC, Dorsolateral prefrontal cortex, Cognitive training

## Abstract

**Background:**

Cognitive models of mood disorders emphasize a causal role of negative affective biases in depression. Computational work suggests that these biases may stem from a belief that negative events have a higher information content than positive events, resulting in preferential processing of and learning from negative outcomes. Learning biases therefore represent a promising target for therapeutic interventions. In this proof-of-concept study in healthy volunteers, we assessed the malleability of biased reinforcement learning using a novel cognitive training paradigm and concurrent transcranial direct current stimulation (tDCS).

**Methods:**

In two studies, young healthy adults completed two sessions of negative (n = 20) or positive (n = 20) training designed to selectively increase learning from loss or win outcomes, respectively. During training active or sham tDCS was applied bilaterally to dorsolateral prefrontal cortex. Analyses tested for changes both in learning rates and win- and loss-driven behaviour. Potential positive/negative emotional transfer of win/loss learning was assessed by a facial emotion recognition task and mood questionnaires.

**Results:**

Negative and positive training increased learning rates for losses and wins, respectively. With negative training, there was also a trend for win (but not loss) learning rates to decrease over successive task blocks. After negative training, there was evidence for near transfer in the form of an increase in loss-driven choices when participants performed a similar (untrained) task. There was no change in far transfer measures of emotional face processing or mood. tDCS had no effect on any aspect of behaviour.

**Discussion and Conclusions:**

Negative training induced a mild negative bias in healthy adults as reflected in loss-driven choice behaviour. Prefrontal tDCS had no effect. Further research is needed to assess if this training procedure can be adapted to enhance learning from positive outcomes and whether effects translate to affective disorders.

**Electronic supplementary material:**

The online version of this article (10.1007/s10608-020-10146-9) contains supplementary material, which is available to authorized users.

## Introduction

Cognitive models of mood disorders emphasise a causal role of negative affective biases in depression and anxiety (Beck 1967; Clark and Beck 2010). Negative biases are expressed as an increase in attention to, encoding of, and memory retrieval for adverse information relative to positive information, which can result in low mood and related depressive symptoms (Dalgleish and Watts 1990; Peckham et al. 2010). Due to their fundamental contribution to depressive symptomatology, there has been increasing interest in the development of interventions aiming to alter affective biases. An influential approach is the use of cognitive training paradigms to modify biased emotional processing directly. The most prominent example is Attentional Bias Modification (ABM), in which patients are asked to respond to a visual probe placed behind either a positive or a negative stimulus. By positioning the probe consistently behind the positive stimulus, people learn to focus on positive rather than negative information. However, evidence for the efficacy of ABM is mixed (Jones and Sharpe 2017; Mogg et al. 2017), with some studies reporting no changes in cognitive bias in multiple versions of ABM (Everaert et al. 2015), small or null effects on depressive symptomatology (Hallion and Ruscio 2011; Mogoaşe et al. 2014), or limited generalization to untrained stimuli (Kruijt et al. 2013). Research on alternative and possibly more efficacious ways to modify affective biases is therefore warranted. In the present study, we investigated the potential of a novel cognitive training paradigm, combined with non-invasive brain stimulation, to induce negative or positive affective biases.

### Computational Mechanisms of Biased Information Processing

A potentially powerful approach to improving symptoms of depression is to target the underlying processes that lead to the *development* of affective biases. Computational models suggest that healthy individuals preferentially process events or outcomes that they estimate to have a high information content (Behrens et al. 2007; Browning et al. 2015; Nassar et al. 2012). Estimated information content can be defined as the extent to which an individual believes that knowledge of a particular outcome will improve their predictions of future outcomes. Crucially, it has been proposed that in depression and anxiety people may *over*-estimate the information content of negative outcomes, which may underpin negative bias and contribute towards symptoms (Pulcu and Browning 2017, 2019). People’s estimates of the information content of events can be inferred from their learning rates. Outcomes that are believed to be highly informative lead to greater updating of expectations and therefore faster learning. Importantly, it has been demonstrated that higher learning rates facilitate memory consolidation and retention across various domains (Abend et al. 2013; Zerr et al. 2018). Targeting learning rates, by designing reinforcement learning paradigms that aim to reduce learning from negative events and/or increase learning from positive events, could therefore represent a promising novel approach to manipulate information processing biases thought to causally maintain symptoms of anxiety and depression.

### Volatility Modulates Learning Rates

One approach to altering people’s estimates of the information content of events, as reflected by learning rates, is to modify the stability versus volatility of the association between a choice and an outcome (Behrens et al. 2007; Browning et al. 2015; Pulcu and Browning 2017). Volatility is a measure of how changeable probabilistic choice-outcome associations are over time (Yu and Dayan 2005). In highly stable conditions, one unexpected outcome is more likely to reflect noise than to signal a change in the underlying choice-outcome probability structure. In this scenario, after such an improbable outcome, individuals should be slow to adjust their expectations and adopt a low learning rate in order to predict future outcomes accurately. In volatile environments, on the other hand, one surprising outcome could indicate a change in the underlying probabilities. Therefore, individuals should rapidly update their expectations and employ a high learning rate to successfully predict future events (Pulcu and Browning 2019). In line with this hypothesis, it has been demonstrated that people tend to have higher learning rates in volatile versus stable conditions (Behrens et al. 2007). Importantly, Pulcu and Browning (2017) have shown that healthy adults can adjust their learning rates for positive and negative events independently in response to volatility variations using the Information Bias Learning Task (IBLT). In the IBLT, participants are asked to choose one of two visual stimuli, each of which is probabilistically associated with a monetary win and loss outcome. Across task blocks, the volatility of these associations is varied to manipulate the relative information content of the win and loss outcomes. Here, we modified the IBLT to develop a novel training paradigm. Specifically, we selected those blocks from the IBLT which either encouraged learning from negative outcomes (i.e., volatile losses) or learning from positive outcomes (i.e., volatile wins). We hypothesised that repeated exposure to one of these block types would selectively increase healthy individuals’ estimates of the information content of negative or positive events, respectively. The goal was to test whether this novel training approach could indeed induce affective bias in healthy adults.

### Augmenting Cognitive Training with tDCS

In addition, we aimed to test whether this novel cognitive training procedure could potentially be enhanced further by concurrently stimulating brain regions causally implicated in negative affective bias (Ironside et al. 2019; Ironside et al. 2016). Transcranial direct current stimulation (tDCS) is a neuromodulatory technique under clinical investigation as a potential treatment for depression (Moffa et al. 2019; Shiozawa et al. 2014). During tDCS a weak electric current of 1–2 mA is delivered through electrodes placed on the scalp. During stimulation, tDCS changes cortical excitability by altering resting membrane potentials, with anodal increasing and cathodal decreasing spontaneous neuronal firing (Nitsche et al. 2003; Nitsche and Paulus 2000; Stagg and Nitsche 2011). Excitability changes outlast the stimulation period, an NMDA receptor dependent effect that reflects stimulation-induced changes in synaptic efficacy (Liebetanz et al. 2002; Nitsche et al. 2003). Evidence from animal and human studies indicates that anodal (excitatory) tDCS can enhance activity-dependent synaptic plasticity (Fritsch et al. 2010). Behaviourally, this has been shown to stabilize learning effects, leading to long-term retention of what is learned (Reis et al. 2009). This suggests that applying tDCS during cognitive training could potentially augment learning effects, which would transfer into therapeutic benefits in clinical populations (O’Shea et al. 2017). At present, the majority of evidence supporting this hypothesis has stimulated the motor system and used tasks of motor function (Buch et al. 2017). Evidence from cognitive studies is more mixed (Dedoncker et al. 2016). A number of studies indicate that tDCS may have synergistic effects on cognitive tasks and depressive symptoms. For example, in healthy adults concurrent anodal tDCS of the left dorsolateral prefrontal cortex (DLPFC) and ABM training was associated with greater changes in the trained direction (either towards or away from threatening stimuli) compared to sham stimulation (Clarke et al. 2014). Similarly, in Major Depressive Disorder tDCS targeting the DLPFC boosted the effects of cognitive control training on mood symptoms (Brunoni et al. 2014) and resulted in greater maintenance of improvements of depressive symptoms post-intervention (Segrave et al. 2014). Applying tDCS concurrent with cognitive training may therefore improve the extent and durability of behavioural outcomes related to affective disorders. Hence we aimed to test this by combining tDCS with our novel cognitive training paradigm.

### Neural Substrates of Learning Rates

The potential efficacy of concurrent tDCS-training paradigms depends heavily on the identification and effective engagement of a suitable neural target for stimulation. Neuroimaging research suggests that negative information processing biases and depression are associated with hypoactivity of the left DLPFC and, often, concurrent hyperactivity of the right DLPFC (Disner et al. 2011; Grimm et al. 2008). Clinical trials have demonstrated that rebalancing DLPFC activity with tDCS, by placing the anode over the left DLPFC and positioning the cathode over the right DLPFC, can improve cognitive functioning and depressive symptoms (Brunoni et al. 2011; Brunoni et al. 2016; Ferrucci et al. 2009). Bilateral DLPFC is therefore a logical stimulation target to combine with cognitive bias modification training. Moreover, the DLPFC is part of distributed brain circuitry critical for reward processing (Haber and Knutson 2010) and reinforcement learning (Lee et al. 2012; Massi et al. 2018). It has been suggested that engagement of the DLPFC during reward learning tasks varies according to the volatility of the association being learned. For example, in a study of probabilistic reward learning in rhesus monkeys, signals in the DLPFC relating to the value of a stimulus were stronger in volatile than non-volatile blocks. Furthermore, in volatile conditions, neurons were found to exhibit a stronger representation of the stimulus chosen on the previous trial if that choice had resulted in a reward compared to no reward (Massi et al. 2018). In addition, it has been shown that the encoding strength of reward magnitude in the DLPFC is greater in volatile than stable conditions in both monkeys and humans, leading to increased weighting of reward magnitude relative to reward probability (Farashahi et al. 2019). Together, these studies provide evidence for a key role of the DLPFC in flexible adaptation of learning to environmental volatility changes. It can therefore be hypothesised that stimulation of the DLPFC could enhance the behavioural effects resulting from volatility shifts in the Information Bias Learning Task.

### Current Study

In the present proof-of-concept study, we tested the effects of cognitive training combined with tDCS on learning rates for positive and negative information in healthy adults. The choice for a healthy participant group was motivated by our primary aim to investigate the *malleability* of learning rates with training and/or tDCS. The presence of a negative bias at baseline, as would be expected in mood disorders, would make direct comparisons of positive and negative training effects challenging. From an ethical and pragmatic perspective, this initial assessment in healthy volunteers is valuable for exploring the parameter space of both training and tDCS protocols relatively quickly, which can then be narrowed down and optimised for future studies in more vulnerable clinical populations. In this study, effects of negative and positive cognitive training were examined in two independent groups, with active versus sham tDCS being compared within-participants in a double-blind design. In the negative training, to increase learning from negative outcomes, IBLT blocks were manipulated such that loss outcomes were volatile (i.e., informative) whereas win outcomes were stable (i.e. uninformative). In the positive training paradigm, the volatility of the win and loss outcomes was reversed to encourage learning from rewards. From a clinical perspective, the rationale and ultimate goal of training and/or tDCS interventions is for positive effects to transfer and improve negative affect. We therefore also examined potential far transfer effects with the Facial Expression Recognition Task (FERT; Harmer et al. 2001), which measures response times and accuracy in identifying ambiguous positive and negative facial expressions. Aberrant performance on the FERT has consistently been observed in depression, with patients presenting with difficulties in accurately identifying emotions (Anderson et al. 2011), with slower responses to sad faces (Gollan et al. 2008), and with less frequent interpretation of neutral faces as being happy compared to healthy controls (Douglas and Porter 2010; Gollan et al. 2008; Surguladze et al. 2004). The FERT has been used extensively to characterise the effects of pharmacotherapeutic interventions on affective biases in both healthy adults and people with depression (Bhagwagar et al. 2004; Browning et al. 2007; Harmer et al. 2003; Tranter et al. 2009). We therefore chose the FERT to test for potential negative/positive emotional transfer after IBLT training because it is a well-validated instrument used in experimental medicine studies with healthy and depressed volunteers. If negative and/or positive training induces valenced information processing biases that generalize beyond the IBLT task, this would be reflected in an increased rate of negative/positive classifications of ambiguous facial expressions, respectively. Overall, we tested the following hypotheses: (1) negative and positive training versions of the IBLT would increase learning rates for losses and wins, respectively; (2) tDCS targeting dorsolateral prefrontal cortex would enhance the effects of the training; (3) alterations to learning rates following combined tDCS/training would transfer to a test of affective processing (the FERT); and (4) behavioural effects of training alone, or training combined with neurostimulation, might generalize to influence acute mood in the training-congruent direction.

## Methods

### Participants

Participants were recruited from the community through flyers posted on local news boards and online advertisements, including university newsletters. Twenty healthy volunteers (4 female, mean age = 23.05, SD ± 6.05) participated in Study 1 (‘loss-volatile’ training). Twenty healthy volunteers (8 female, mean age = 24.45, SD ± 5.06) participated in Study 2 (‘win-volatile’ training). Exclusion criteria were a history of psychiatric disorders, neurological illness, use of psychoactive medication, personal or family history of epileptic fits or seizures, and any contraindications to tDCS. Written informed consent was obtained from all volunteers and study procedures were approved by the local ethics committee (CUREC; R48995/RE003) and performed in accordance with the 1964 Helsinki declaration and its later amendments.

### Study Procedure

**Fig. 1:**
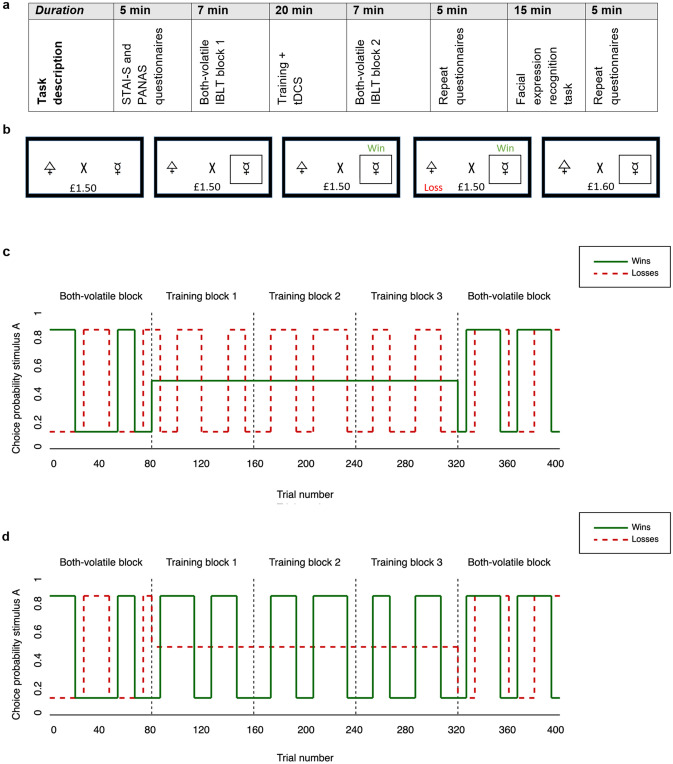
Schematic overview of the training sessions. **a** Timeline study procedure. **b** Example of a trial on the Information Bias Learning Task (IBLT). At the beginning of the task, participants are provided with a start amount of £1.50. In each trial, a fixation cross flanked by two abstract stimuli is presented and the participant has to choose one of the stimuli via a button press. Once a stimulus is chosen, a win and a loss outcome are presented consecutively, with the order of their appearance (win first versus loss first) being randomised across trials. If the chosen stimulus is associated with a win the participant gains 10p, and if the chosen stimulus is associated with a loss the participant loses 10p. If the win and loss outcome both appear over the same stimulus, the participant does not win or lose any money irrespective of their choice. The win and loss outcomes are independent, meaning that the location of the win does not provide any information about the location of the loss. In this task, participants have to learn through experience which stimulus to choose in order to maximise total winnings. **c** Structure of the IBLT for negative training in Study 1. The task consisted of 5 blocks comprised of 80 trials each (vertical, dashed black lines separate the individual blocks). The x-axis represents the number of trials, with the y-axis indicating the probability *p* of an outcome appearing over stimulus ‘A’. The probability of the outcome appearing over stimulus ‘B’ can be calculated as 1 − *p*. The win outcomes are represented as continuous green lines, with the loss outcomes corresponding to the dashed red lines. The volatility of the win and loss outcomes is manipulated across the task blocks, with higher volatility being associated with a higher information content. In the first block, both the wins and losses are volatile (‘Both-volatile’ block), with the probability of an outcome appearing over stimulus ‘A’ switching between 20 and 80%. Here, both outcomes have a high information content, such that if the win/loss appears over shape ‘A’, it is more likely to be associated with shape ‘A’ than shape ‘B’ in the subsequent trials. In this block, participants are therefore expected to have high learning rates for both wins and losses. In blocks 2–4, on the other hand, volatility is manipulated so that losses are highly informative and wins are uninformative (‘Training’ blocks). Whereas the loss outcomes remain volatile, the association of shape ‘A’ with the win outcome is stable at 50%. Thus, the chance of the win appearing over either of the shapes remains equal across the trials, with its location on one trial providing no information about future trials. In these ‘Training’ blocks, it is expected that participants will have higher learning rates for loss than win outcomes. Finally, block 5 consists of another ‘Both-volatile’ block, in which both wins and losses are volatile. By comparing learning rates in block 5 with block 1, it is possible to quantify potential shifts in learning from win and loss outcomes following the ‘Training’ blocks. **d** Structure of the IBLT for positive training in Study 2. Similar to Study 1, the IBLT is comprised of a ‘Both-volatile’ block, three ‘Training’ blocks, and a final ‘Both-volatile block. However, volatility of the win and loss outcomes in the ‘Training’ blocks is reversed, such that win outcomes are highly informative (volatile) and loss outcomes are uninformative (stable). Therefore, contrary to Study 1, participants are expected to demonstrate higher learning rates for win than loss outcomes in the ‘Training’ blocks of Study 2 (Color figure online)

In both Study 1 and Study 2, participants were invited to two cognitive training sessions which were carried out at least one week apart (see Fig. 1a). In one session participants received sham tDCS, whereas in the other session they were given active tDCS of bilateral DLPFC. The order of tDCS conditions was counterbalanced and double-blinded in both studies.

### IBLT Training

The computerised Information Bias Learning Task (IBLT) training paradigm was adapted from reinforcement learning tasks previously used to investigate effects of volatility on win and loss learning rates (Behrens et al. 2007; Browning et al. 2015; Pulcu and Browning 2017). The IBLT training consisted of 5 blocks of 80 trials each, with a brief 30 s rest between each of the blocks. On every trial, participants were presented with a fixation cross in the centre of the screen flanked by two abstract shapes (letters selected from the Agathodaimon font) offset by approximately 7° visual angle. Participants were instructed to press a button to choose between the two visual stimuli, which were probabilistically associated with a win and a loss outcome. Details of the format and stimuli utilised in the training task are provided in Fig. 1b. The win and loss outcomes were independent, such that the association of a win outcome with a stimulus did not determine the association of the loss outcome and vice versa. Predicted stimulus-outcome associations were learned over time through experience on previous trials. The volatility of stimulus-outcome contingencies of wins and losses was modulated across the task blocks. In a volatile state, the association of stimulus ‘A’ and a win (or loss) outcome switched between 20 and 80% in runs of 14–30 trials. In stable conditions, the association between stimulus ‘A’ and a win (or loss) outcome remained constant at 50%. Within each of the five task blocks the same two shapes were used for all trials, with different shapes being used between the task blocks.

In Study 1, participants completed three negative ‘Training’ blocks, in which loss outcomes were volatile while win outcomes were stable (see Fig. 1c). In Study 2, participants carried out three positive ‘Training’ blocks where the win outcomes were volatile and loss outcomes were stable (see Fig. 1d). The ‘Training’ blocks took approximately 21 min to complete in each study, just outlasting the duration of concurrent tDCS (20 min). In both Study 1 and Study 2, participants also completed a ‘Both-volatile’ block directly before and after the three ‘Training’ blocks. In ‘Both-volatile’ blocks, both the win and loss associations were volatile (i.e. informative), which was expected to lead to high learning rates for both outcome types. The first ‘Both-volatile’ block, which took place before the ‘Training’ blocks, was used as a measure of baseline learning rates for informative wins and losses. The final ‘Both-volatile’ block, which was completed after the ‘Training’ blocks, was included to compare learning rates before and after training. A shift in learning rates in ‘Both-volatile’ blocks after training could be interpreted as a near transfer effect of the training. For instance, negative training in Study 1 was designed to induce higher learning rates from losses and/or lower learning rates from wins. Near transfer of this learning would be reflected in a corresponding increase in loss learning rates in the ‘Both-volatile’ block after training compared to before training. Similarly, near transfer from the positive training in Study 2 was expected to lead to higher learning rates from wins and/or lower learning rates from losses, with transfer reflected in an increase in win learning rates in the ‘Both-volatile’ block after training compared to before training.

### tDCS Protocol

Stimulation was delivered during the three IBLT training blocks using a battery-powered device (Eldith DC-Stimulator Plus, Neuroconn, Germany). The electrodes (5 × 5 cm) were placed in saline-soaked sponges and attached to the scalp using rubber bands. The anodal electrode was placed on the left DLPFC, while the cathodal electrode was placed over the right DLPFC (F3 and F4, respectively, according to the 10/20 system of electrode placement). In the active tDCS condition, stimulation was delivered at 2 mA for 20 min, with 10 s ramping-up and ramping-down. In the sham tDCS condition, participants received 30 s of direct current followed by impedance control with a small current pulse being produced every 550 ms (110 µA over 15 ms), resulting in an instantaneous current of no more than 2 µA.

### Facial Expression Recognition Task

Following the cognitive training and tDCS, participants completed the Facial Expression Recognition Task (FERT). In the FERT, participants are asked to identify positive and negative emotions in ambiguous facial expression stimuli. All emotions are morphed between 0% intensity (neutral expression) and 100% intensity (full negative or positive expression) in 10% increments (see Online Resource 1 for further task details). Due to the ambiguous nature of the stimuli the task can detect potential biases in processing and interpretation of positive and negative facial expressions. The purpose of this task was to assess whether the predicted negative versus positive information processing biases induced by the training/tDCS would transfer to an untrained emotional processing task. The main outcomes of interest were response time, accuracy (correct identification of emotions), and misclassifications (incorrect identification of emotions) for each expression. Far transfer effects would be reflected in a change in reaction times and/or accuracy in identifying facial expressions congruent with the training valence.

### Questionnaire Measures

In order to gain quantitative measures of participants’ trait and state mood and anxiety levels, questionnaires were completed throughout the training sessions. At the beginning of the first session, participants completed Beck’s Depression Inventory II (BDI-II) (Beck et al. 1996) and the Trait subscale of the State-Trait Anxiety Inventory (STAI-Trait) (Spielberger et al. 1983). In addition, participants filled out the State version of the State-Trait Anxiety Inventory (STAI-State) (Spielberger et al. 1983) and the Positive and Negative Affect Scales (PANAS) (Watson et al. 1988) before the IBLT task, after the IBLT task, and after completion of the FERT (see Fig. 1a).

### Computational Modelling

To estimate learning parameters on the IBLT blocks, a computational model identical to that previously applied by Pulcu et al. (2019) was developed using MATLAB (R2016B, The Mathworks Inc, Natick, MA). In this model, separate learning rates were estimated for the loss and win outcome to assess learning from negative versus positive information. In addition, values were estimated for the ‘inverse temperature’, a parameter which represents the randomness or stochasticity of participants’ choices (i.e., how closely do participants’ choices correspond with the estimated value of the stimuli?). That is, individuals with a high inverse temperature value tend to choose the option that they believe is most likely to result in the best possible outcome. In contrast, an inverse temperature of 0 indicates that the participant is equally likely to choose any of the available options, independent of their likelihood of resulting in a positive outcome. Model selection was based on a comparison of six different computational models using Akaike Information Criterion (AIC) and Bayesian Information Criterion (BIC) values. The AIC and BIC quantify the relative performance of different models by comparing their goodness of fit to the data and parsimony, favouring models with fewer parameters (Vrieze 2012). Out of the six models assessed, the model used here was found to have the lowest AIC and BIC scores, indicating best performance. A description of each model and corresponding AIC/BIC values is provided in Online Resource 1.

First, the model calculates the probability estimates that the win and loss outcomes would be associated with shape “A” using a Rescorla–Wagner learning rule (Rescorla and Wagner 1972):

1 $${rwin}_{(i+1)}={rwin}_{(i)}+ {\alpha \mathrm{w}in* \varepsilon }_{win(i)}$$

$${rloss}_{(i+1)}={rloss}_{(i)}+ {\alpha loss* \varepsilon }_{loss(i)}$$

in which *rwin*_(*i*+1)_ is the estimated win outcome probability for the *i* + 1st trial, *rwin*_(*i*)_ is the estimated outcome for the *i*th trial, *αwin* represents the learning rate for win outcomes, and *ε*_*win*(*i*)_ indicates the prediction error on the *i*th trial. Prediction error is calculated as the predicted outcome value minus the actual outcome value. At the start of each block, *rwin* was initialised at 0.5 because participants could not have prior expectations about which shape was most likely to be associated with a win outcome. *rloss*_(*i*+1)_, *rloss*_(*i*)_, *aloss* and *ε*_*loss*(*i*)_ indicate the same variables for the loss outcome. Next, estimated outcome probabilities were transformed into a single choice probability using a softmax function:

2 $${P}_{(choice=A(i))}= \frac{1}{1+{\mathrm{exp}}^{(-{\beta *(rwin}_{(i)}+t-{rloss}_{(i)}))}}$$

Here, *P*_(*choice*=*A*(*i*))_ is the probability of choosing shape A in trial *i*, with *β* representing the inverse decision temperature and *t* reflecting an added parameter used to estimate a general tendency to select one of the options over the other. The inverse temperature indicates the degree to which the expected values are used to determine choice for a particular shape. Learning rates and *β*-values were calculated separately for each task block and participant. This was achieved by calculating the full joint posterior probability of the parameters given participants’ choices, deriving the expected value of each parameter from their marginalised probability distributions (Behrens et al. 2007; Browning et al. 2015). As the purpose of this model was to measure change in learning rates between blocks rather than describe the mechanism by which estimated information content is calculated, it was fit separately to each of the task blocks. The first 10 trials of each block were omitted when fitting the model parameters to participants’ choices, as initial learning rates are generally inflated due to estimation uncertainty during new tasks.

### Win- and Loss-Driven Behaviour

**Fig. 2:**
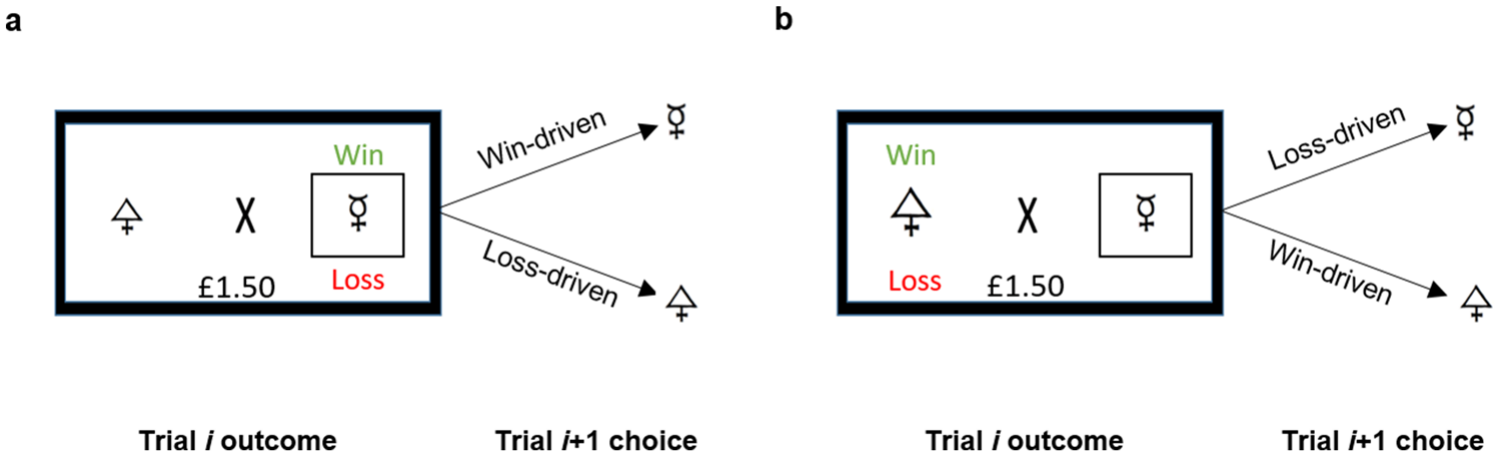
Schematic illustration of win-driven behaviour on the IBLT. **a** If the participant had chosen a stimulus associated with both a win and a loss on trial *i*, choosing the same stimulus on the next trial *i* + 1 suggested a stronger impact of win than loss outcomes on behaviour. **b** Conversely, if the participant had chosen a stimulus with neither a win nor a loss on trial *i*, selecting the alternative stimulus on the next trial *i* + 1 indicated a greater influence of win than loss outcomes on choice behaviour

To complement the model-based analyses, non-computational analyses were conducted to examine win- and loss-driven behaviour following trials where a shape was associated with *both* a win and a loss (Pulcu et al. 2019). The reason for including trials of this type was to obtain a measure of which outcome had a greater influence on participants’ future choices that did not depend on the assumptions of a particular model. If one stimulus was associated with both a win and a loss on trial *i*, the participant would be expected to select this stimulus in the next trial (*i* + 1) if they are influenced more by the win outcome. Conversely, if they are more influenced by the fact that a loss outcome is associated with a stimulus in trial *i*, then they should select the stimulus that had neither a win nor a loss on the next trial (*i* + 1). The stimulus chosen by the participant on trial *i* + 1 therefore provides a measure of participants’ beliefs about the relative informativeness of positive versus negative outcomes, indicated by which has a stronger impact on their subsequent choice behaviour (see Fig. 2). Win-driven choice behaviour was quantified as the sum of the following choices:

- If the stimulus on trial *i* had both a win and a loss, participants selected this stimulus on trial *i* + 1
- If the chosen stimulus on trial *i* had neither a win nor a loss, participants selected the other stimulus on trial *i* + 1

The proportion of win-driven choices was then calculated by dividing the number of win-driven choices by the total amount of trials on which a stimulus was associated with both a win and a loss (40 per block). The proportion of loss-driven choices in turn can be derived from the win-driven choice behaviour (i.e. 1 − proportion win-driven choices).

### Statistical Analyses

All statistical analyses were carried out in R software (R Version 3.6.0.). Data and analysis scripts are available on Open Science Framework: https://osf.io/k36h4/, DOI: 10.17605/OSF.IO/K36H4. Changes in learning rates and inverse temperature were examined using repeated-measures ANOVAs using the ‘ezANOVA’ function from the ‘ez’ R package. For all IBLT blocks, within-subject variables were Outcome valence (win vs. loss), and tDCS condition (active vs. sham). In addition, a within-subject variable for Block (1, 2, or 3) was included for ‘Training’ blocks and Time (before or after training) for ‘Both-volatile’ blocks. tDCS order (sham first vs. active first) was included as a between-subject variable. Inverse temperature values were similarly investigated using Block/Time, tDCS condition, and tDCS order as predictor variables. Parameters were transformed onto the infinite real line using a logit transformation for learning rates and a log transformation for the inverse temperature values, in line with previously published work by Pulcu and Browning (2017). Figures and reported values represent raw parameter values to facilitate interpretation of the results. To account for individual differences in baseline learning parameters, all analyses were also run including baseline learning rates from the first ‘Both-volatile’ block of the first testing session as covariates. All results were unchanged when baseline learning rates were included in the analyses.

Accuracy, Reaction time, and Misclassifications on the FERT were investigated in repeated-measures ANOVAs. The predictor variables were Emotion (Anger, Disgust, Fear, Happiness, Sadness, and Surprise), tDCS condition, and tDCS order. For direct comparison of task performance between the two studies, Study (1 vs. 2) was added as a between-subject factor.

Alterations in acute mood and anxiety were investigated based on responses on the PANAS and STAI-State questionnaires. Effects of stimulation and training were examined with Time (before IBLT, after IBLT, or after FERT completion), tDCS condition, and tDCS order as predictor variables for changes in PANAS Positive, PANAS Negative, and STAI-State scores.

## Results

### Baseline Questionnaire Measures

Baseline scores on the mood questionnaires for Study 1 and Study 2 are provided in Table 1. Scores on the BDI-II and STAI-Trait suggest that participants in both studies had few symptoms of depression or trait anxiety. Prior to tDCS/training, there were no significant differences in state mood questionnaire scores (PANAS Positive, PANAS Negative, and STAI-State) between the active and tDCS sessions in either Study 1 or Study 2 (all *p* > 0.05), indicating that there was no systematic difference in baseline mood between the study sessions.

**Table 1:** Mean (SE) baseline questionnaire scores for participants completing negative IBLT training (Study 1) and positive IBLT training (Study 2)

	Study 1 Negative IBLT training	Study 2 Positive IBLT training
BDI-II	3.70 (1.33)	4.00 (33.15)
STAI-Trait	34.35 (2.08)	33.15 (1.22)
PANAS Positive		
Sham tDCS session	33.40 (1.39)	32.35 (1.70)
Bifrontal tDCS session	33.45 (1.26)	33.60 (1.87)
PANAS Negative		
Sham tDCS session	11.55 (0.62)	11.35 (0.43)
Bifrontal tDCS session	10.50 (0.18)	11.80 (0.63)
STAI-State		
Sham tDCS session	27.40 (1.52)	26.15 (1.29)
Bifrontal tDCS session	26.35 (1.36)	26.60 (1.15)

*BDI-II* Beck’s Depression Inventory II, *PANAS* Positive and Negative Affect Scale, *STAI* State-Trait Anxiety Inventory

### Study 1: Effects of Negative IBLT Training and Concurrent tDCS on Reward Learning

#### Computational Learning Parameters

**Fig. 3:**
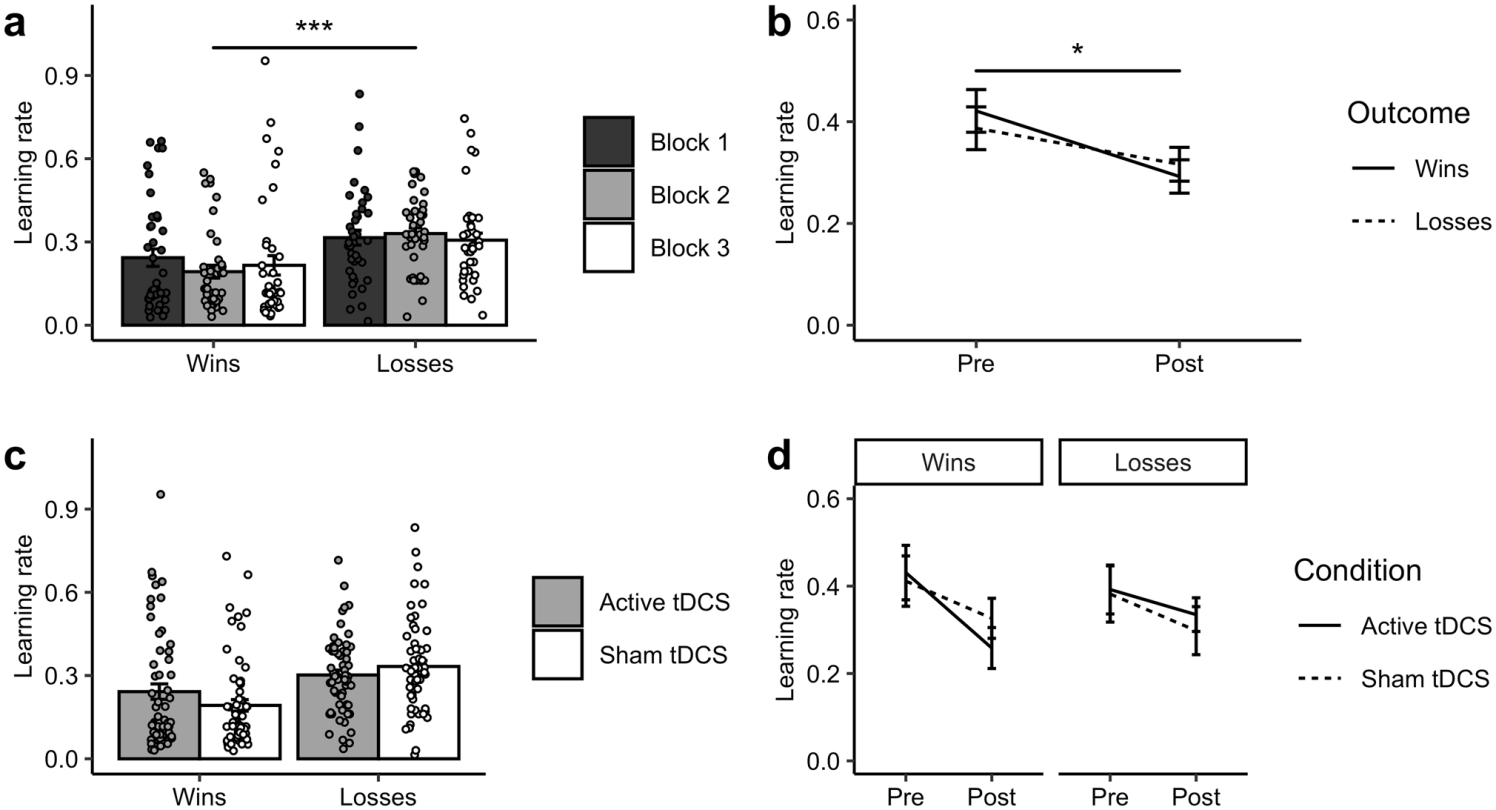
Effects of negative IBLT training and tDCS on learning rates. **a** Across the three ‘Training’ blocks, participants demonstrated significantly higher learning rates for negative (loss) than positive (win) outcomes. Learning rates were pooled over tDCS condition. **b** Learning rates for both win and loss outcomes decreased over time in the ‘Both-volatile’ blocks carried out before (‘Pre’) and after (‘Post’) the training. Learning rates were pooled over tDCS condition. **c** Active tDCS did not alter learning rates for either wins or losses in the ‘Training’ blocks compared to sham tDCS. Learning rates are averaged across the three ‘Training’ blocks. **d** In the ‘Both-volatile’ blocks there were no significant effects of tDCS (*p* > 0.05) on learning rates for either wins or losses over time when contrasting blocks completed before (‘Pre’) and after (‘Post’) training. This suggests that tDCS did not influence the near-transfer of training effects on speed of learning. *p < 0.05, ***p < 0.001

The aim of the negative IBLT training paradigm was to induce an affective bias towards learning from negative (‘loss’) outcomes. In the ‘Training’ blocks, it was expected that participants would adopt higher learning rates for the volatile loss outcomes than for the stable win outcomes. Confirming the effectiveness of the training manipulation, participants had a higher learning rate for losses than wins in the ‘Training’ blocks (*F*(1,18) = 24.22, *p* < 0.001, η^2^ = 0.112). It was also predicted that, through gaining experience on the task, learning rates for loss outcomes would increase over time. Contrary to this hypothesis, however, there was neither a main effect of Block (*F*(2,36) = 0.30, *p* = 0.746) nor an interaction of Block and Outcome valence (*F*(2,36) = 1.62, *p* = 0.211). As shown in Fig. 3a, a negative learning bias was rapidly induced in ‘Training’ block 1 and persisted in the subsequent two blocks.

The key hypothesis was that negative training would transfer to the ‘Both-volatile’ blocks, reflected in a shift to faster learning from losses than wins after training compared to before. We observed a decrease in learning rates over time (*F*(1,18) = 5.78, *p* = 0.027, η^2^ = 0.022), with learning rates being higher (for both wins and losses) before than after IBLT training. Importantly, however, there was also a numerical trend towards an interaction of Time with Outcome valence (*F*(1,18) = 3.51, *p* = 0.077, η^2^ = 0.008), with average learning rates decreasing more for win (*t*(39) = 2.79, *p* = 0.008) than loss outcomes (*t*(39) = 0.64, *p* = 0.525) over time (see Fig. 3b). This trend is consistent with the aim of the training procedure to encourage faster learning from negative relative to positive outcomes.

The purpose of applying active tDCS was to test if this would enhance the expected effects of IBLT training, potentially causing a greater increase in loss learning rates in the ‘Training’ blocks compared to sham stimulation, which could also manifest in enhanced transfer. However, in the ‘Training’ blocks (see Fig. 3c) there was neither a main effect of tDCS (*F*(1,18) = 0.01, *p* = 0.916) and no interaction with Outcome valence (*F*(1,18) = 0.03, p = 0.873). Similarly, in the ‘Both-volatile’ blocks (see Fig. 3d) there was neither an interaction of tDCS with Time (*F*(1,18) = 0.02, *p* = 0.903) nor a 3-way interaction with Time and Outcome valence (*F*(1,18) = 1.04, *p* = 0.321). Overall, there was thus no evidence that tDCS of the DLPFC altered learning rates or transfer in this study.

Finally, it was expected that the IBLT training/tDCS manipulation would be specific to learning rates and would not affect other computational parameters. Consistent with this, there was no change in the randomness of participants’ choices as a function of Block/Time, tDCS or Outcome valence (inverse temperature values all *p* > 0.05). This shows that IBLT training specifically altered participants’ learning rates without altering other aspects of their behaviour (e.g. choice stochasticity).

#### Win- and Loss-Driven Choice Behaviour

**Fig. 4:**
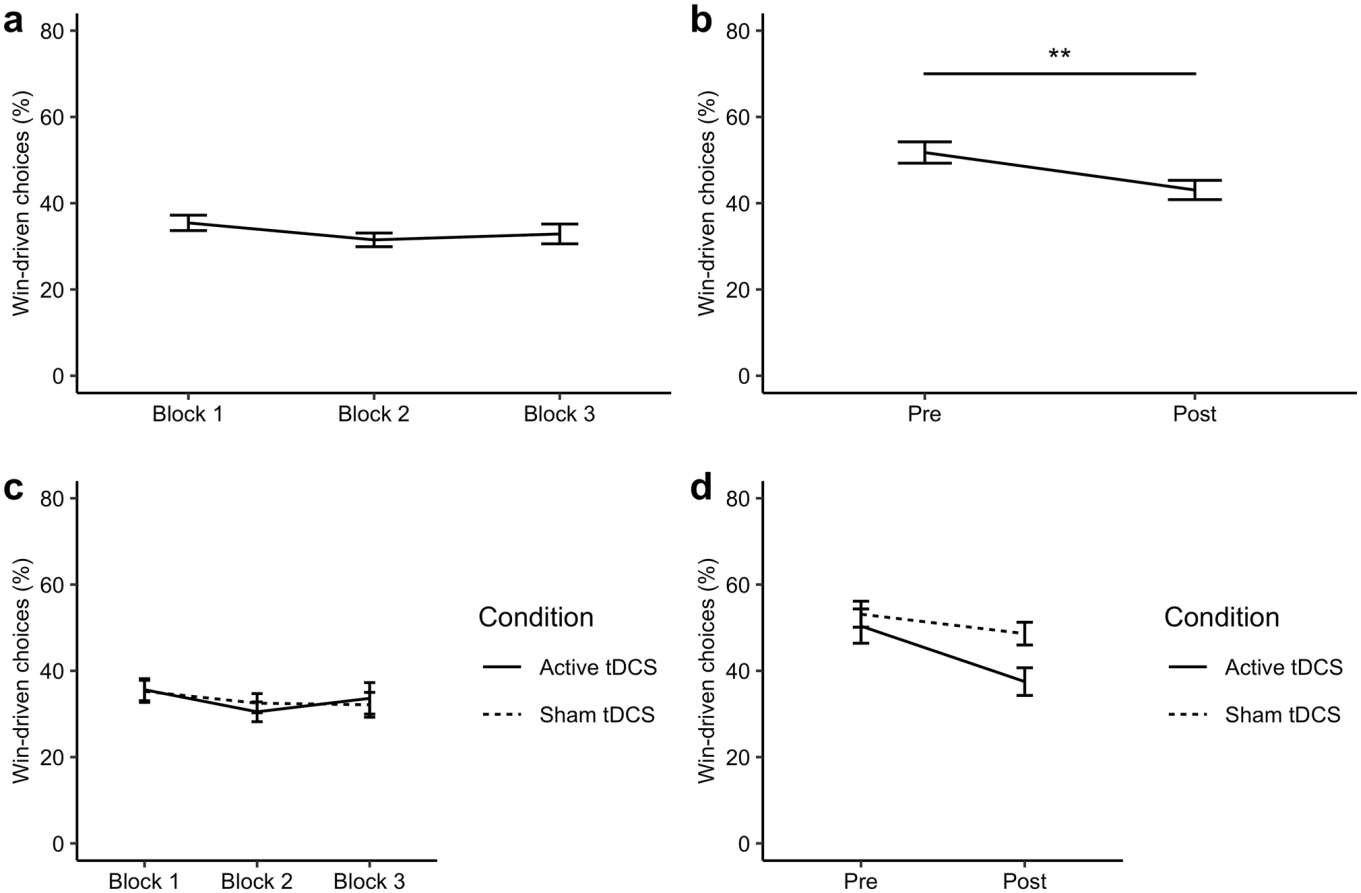
Proportion (*p*) of win-driven choices during negative IBLT training by tDCS condition. The proportion of loss-driven choices can be calculated as 1 − *p*. **a** In the ‘Training’ blocks, participants tended to make more loss- than win-driven choices across the three blocks. **b** There was a significant decrease in win-driven choices (i.e. an increase in loss-driven choices) over time in the ‘Both-volatile’ blocks completed before (‘Pre’) and after (‘Post’) training. There was no evidence for an effect of tDCS in either the **c** ‘Training’ blocks or the **d** ‘Both-volatile’ blocks. **p < 0.01

As the negative IBLT training fostered learning from loss outcomes, it was expected that the overall number of loss-driven choices would be greater than the number of win-driven choices in the ‘Training’ blocks. Demonstrating that negative IBLT training successfully induced a bias towards basing choices on losses, the loss-driven option was selected over the win-driven option in 66.73% of the trials across the ‘Training’ blocks (see Fig. 4a).

Our key prediction for the ‘Both-volatile’ blocks was that IBLT training would result in an increase in loss-driven choices, such that participants would choose the loss-driven option more frequently after than before training. A repeated-measures ANOVA confirmed that participants made a significantly greater number of loss-driven choices after completing the ‘Training’ blocks compared to baseline performance (*F*(1,18) = 10.06, *p* = 0.005, η^2^ = 0.090) as shown in Fig. 4b. This increase in loss-driven choices after negative IBLT training is consistent with the trend towards near-transfer for loss learning rates.

Finally, tDCS of the DLPFC was expected to further increase the number of loss-driven choices compared to sham stimulation. However, there was again no evidence for modification of IBLT performance with tDCS (see Fig. 4c, d). Specifically, there was no significant main effect of stimulation in the ‘Training’ blocks (*F*(1,18) = 0.00, *p* = 0.987) nor an interaction of tDCS with Time in the ‘Both-volatile’ blocks (*F*(1,18) = 1.79, *p* = 0.198).

#### Emotional Face Recognition

We predicted that combined negative IBLT training/tDCS would result in improved recognition of negative facial expressions on the FERT. In contrast to this hypothesis, however, there was no evidence that either training or tDCS significantly affected recognition of emotional face stimuli (see Online Resource 1).

#### Mood and Anxiety Measures

Finally, we tested whether training towards learning from negative outcomes resulted in acute declines in mood and increased anxiety. Whilst there were no changes in STAI-State scores (*F*(2,38) = 1.38, *p* = 0.265) or PANAS Negative affect scores (*F*(2,38) = 0.12, *p* = 0.884) over time, there was a significant decrease in PANAS Positive affect scores (*F*(2,38) = 9.23, *p* < 0.001, η^2^ = 0.056). Potentially, this change in positive mood could be attributed to the negative IBLT training. Alternatively, it is possible that there may be other non-specific factors (e.g. fatigue, loss of motivation) which contribute to reduced positive affect over time.

Finally, we assessed the effects of tDCS on mood and anxiety when combined with negative IBLT training. There were no main or interaction effects of tDCS for STAI-S, PANAS Positive, or PANAS Negative scores (all *p* > 0.05).

### Study 2: Effects of Positive IBLT Training and Concurrent tDCS on Reward Learning

**Fig. 5:**
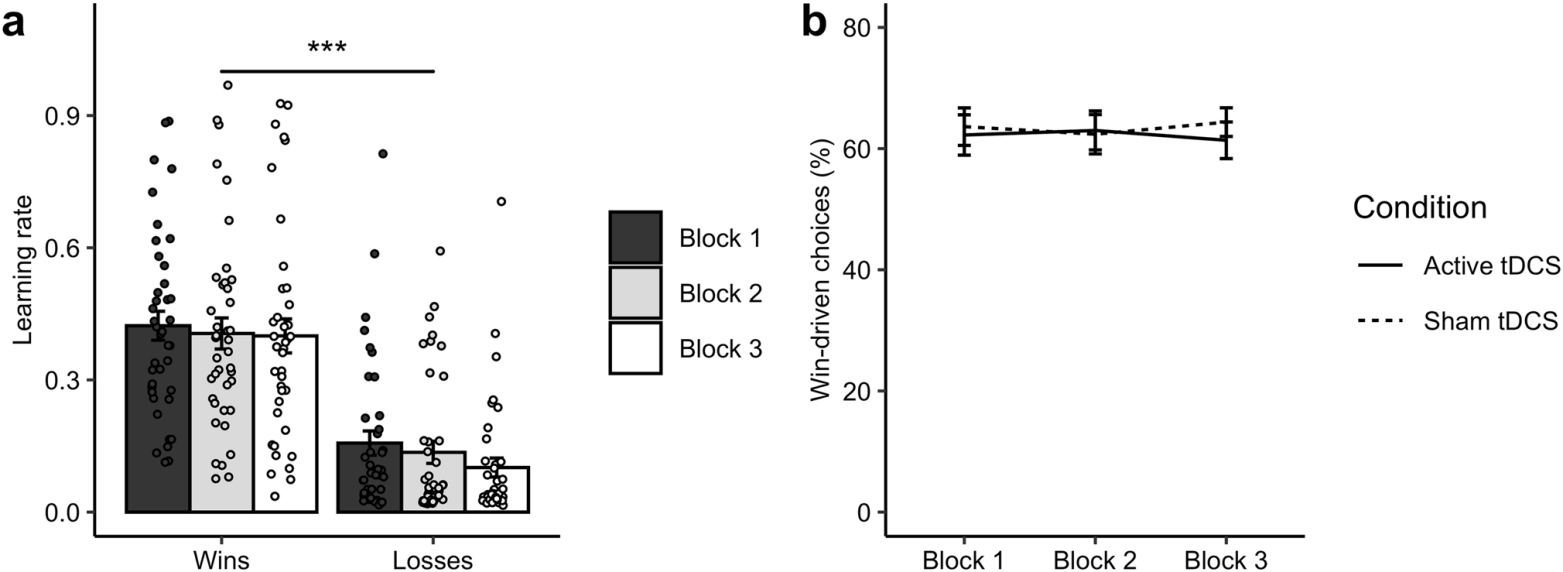
Effects of positive IBLT training on learning rates and win-driven choice behaviour. **a** Across the three ‘Training’ blocks, participants demonstrated significantly higher learning rates for positive (win) than negative (loss) outcomes. **b** On average, the proportion of win-driven choices (*p*) was greater than the proportion of loss-driven choices (1 − *p*) across the ‘Training’ blocks. ***p < 0.001

The positive IBLT training protocol aimed to induce a bias towards learning from positive (‘win’) outcomes. It was expected that the effects of training would be the reverse of the negative IBLT training in Study 1, with training leading to faster learning from win than loss outcomes. Consistent with this, participants demonstrated significantly higher learning rates for positive than negative outcomes on the ‘Training’ blocks (*F*(1,18) = 135.39, *p* < 0.001, η^2^ = 0.452). In addition, non-computational analyses indicated that participants chose the win-driven option over the loss-driven option on 62.8% of the trials. These findings demonstrate that the positive IBLT training effectively induced a congruent learning bias in the ‘Training’ blocks (see Fig. 5). However, we found no evidence of near transfer with positive IBLT training/tDCS for learning rates, win-driven behaviour, FERT performance, or mood (see Online Resource 1).

## Discussion

In the two studies presented here, we investigated the effects of a novel cognitive training paradigm with concurrent bifrontal tDCS on affective learning processes. Our results suggest that training with informative (volatile) wins did not produce a positive affective bias beyond the training blocks. However, we found several lines of evidence supporting the hypothesis that training towards informative (volatile) losses can induce a negative affective bias in healthy adults. First of all, participants demonstrated higher learning rates for loss than win outcomes across the training blocks, highlighting the efficacy of the volatility manipulation in training. Secondly, non-computational analyses demonstrated an increase in loss-driven choices in ‘Both-volatile’ blocks for trials where the win and loss outcome were associated with the same shape. This shows that there was near transfer of learning from losses in the negative IBLT training paradigm. Finally, there was a non-significant trend towards near transfer of changes in learning from training, with a greater decrease in win than loss learning rates being observed in the ‘Both-volatile’ blocks completed after training. This again suggests a relative increase in reliance on loss compared to win outcomes after carrying out the negative IBLT training. Together, these findings indicate that the negative training protocol introduced here was able to influence affective cognition in the intended direction. Contrary to hypothesis, the application of brain stimulation to the DLPFC did not modulate the impact of either negative or positive cognitive training. We discuss the implications of these outcomes and potential research directions for further development of this simultaneous training-tDCS approach.

### Increasing Learning from Negative Outcomes

A key finding of the present research was that training towards negative outcomes resulted in a greater number of loss-driven choices in ‘Both-volatile’ blocks following the training. This increase in loss-driven behaviour was accompanied by a trend towards increased learning from losses relative to wins in the computationally derived learning rates. A possible explanation for the significant effect being observed for loss-driven choices but not learning rates is that analyses of win- and loss-driven choices focused only on a subset of trials—those following immediately after both the win and loss outcome were associated with the same stimulus. The ‘both’ outcome on these trials does not provide objective information about which stimulus would be the optimal choice on the subsequent trial. Hence, these trials provide an indirect measure of participants’ *subjective* estimates of the relative informativeness of the win versus loss outcome information. Participants who believe the win outcome better predicts future success are more likely to select the stimulus which was associated with both outcomes on the subsequent trial. Participants who believe the loss outcome better predicts the future are more likely to select the stimulus which was not associated with either outcome on the next trial. After negative training, participants showed an increase in loss-driven choices, suggesting that the negative training had the desired effect, i.e. it increased participants’ use of negative outcome information to guide their future choices, although this effect was specific to ambiguous situations (i.e. ‘both’ outcome trials). However, it should be noted that the effect size was small, and there was no evidence that changes in negative IBLT training influenced affective bias as measured on a task of recognition of emotional facial expressions. In addition, although there was a reduction in positive affect after negative training, a similar change in mood was observed with positive training which may be due to reduced interest and motivation with time on task. It is therefore unclear whether a single session of negative training can specifically affect mood in healthy volunteers. The significance of this isolated finding as confirmation that our novel training paradigm is an effective form of cognitive bias modification should therefore be interpreted with care. More positively, if this training effect can be replicated, and the general approach modified to also increase positive (and not just negative) resolution of ambiguity, this could have potential application for targeting cognitive symptoms of depression.

The present findings were observed in healthy volunteers. Although there was a main effect of training, with negative training associated with overall greater learning rates for losses versus wins, and positive training associated with overall greater learning rates for wins versus losses, there was no progressive increase in learning rates across successive ‘Training’ blocks 1–3, as might be expected a priori. This suggests a possible ceiling effect in this training paradigm in healthy volunteers, such that learning rates were already maximal in the first exposure block. Notably, the healthy adults taking part in this study had a high learning rate for rewarding outcomes at baseline. This is in line with previous research indicating that healthy individuals tend to have an optimism bias (Sharot 2011), which is associated with greater attention to positive and rewarding stimuli compared with negative or neutral stimuli (Kress et al. 2018; Pool et al. 2016). Given this baseline bias for learning from positive outcomes, there may have been relatively little scope for further enhancing learning from rewards in this group. We speculate that such a positive bias and ceiling effect with positive training is less likely to be present in depressed volunteers, who may therefore have more room for training to change learning rates. Further testing of this paradigm in clinical populations will therefore be essential to determine mood state-dependent effects of the training on learning processes.

We are currently testing this approach in analogue samples of community volunteers with subclinical depression to assess potential effects on learning rates and on ambiguous ‘both-outcomes’ trials. It is well-established that depression is associated with a negative interpretation bias for ambiguous information (Cowden Hindash and Amir 2012; Lawson et al. 2002). For instance, when asked to write down orally presented words that could have a negative, positive, or neutral meaning (e.g. die/dye), people with depression tend to use the spelling of the negative word (Mogg et al. 2006). It has been suggested that this interpretation bias is a result of negative attention biases that in turn can cause congruent bias in memory (Everaert et al. 2014). It can therefore be hypothesised that cognitive training that modifies attention to negative versus positive information in ambiguous contexts could impact on related cognitive biases (e.g. memory). If future work indicates that IBLT training can increase learning from positive outcomes in depressed individuals, a key test of clinical potential will be whether this effect generalizes to other negatively biased cognitive domains such as attention and memory.

### Absence of tDCS Effects on Learning

Several previous studies have reported that tDCS can increase the effects of cognitive training (Brunoni et al. 2014; Clarke et al. 2014; Segrave et al. 2014). However, we did not find a synergistic effect of prefrontal neurostimulation on IBLT training. This was unexpected, as previous studies using the same tDCS parameters have reported significant decreases in affective bias (Ironside et al. 2016) and reduced symptoms of depression (Brunoni et al. 2016). There are several possible explanations. A first possibility is that, as indicated previously, there may be a ceiling effect such that healthy participants rapidly adopt high learning rates for volatile stimuli, which cannot be increased further.

Alternatively, it has been suggested that positive and negative outcomes have different value functions and correspondingly different underlying neural bases (Chen et al. 2015). It is thus possible that modification of loss learning mechanisms requires a different tDCS protocol compared with reward learning. To date, most studies investigating the neural bases of learning with volatile outcomes have focused on reward rather than loss outcomes (Behrens et al. 2007; Massi et al. 2018). Consequently, it is at present unclear whether volatility of loss outcomes is encoded in similar brain circuits as win outcomes. Additional research contrasting responses to reward and loss outcomes in volatile conditions is needed to clarify the neural mechanisms of these affective learning processes.

### Limitations and Future Directions

Our study has several limitations which we plan to address in ongoing and future studies to investigate the potential of IBLT training and tDCS in modifying depression-related biases. First, similar to previous tDCS research, our studies used a relatively small sample size. Since our prime motivation was the potential future clinical utility of training and/or tDCS, our interest was in detecting medium to large effects. Our chosen sample size was informed by prior work (in preparation) using the same task and tDCS protocol but without a training element. There we found a medium effect (d = 0.41) of tDCS on the task, which we replicated, with each experiment involving a sample of 20 participants. It therefore seemed reasonable to choose the same sample size for this proof-of-principle training study. However, we note that relying on prior significant results can inflate the predicted effect size, and thereby hamper estimation of the appropriate sample size for replication studies (see Button et al. 2013). The lack of power to detect small effects in the present work could be addressed in larger replication studies employing formal power calculations.

In addition, interpretations of the present work are limited by the absence of a control condition for cognitive training. First, a ‘sham’ training condition would be useful to disambiguate learning effects from a time or task familiarity effect. Second, the lack of a control training paradigm meant that we were unable to examine the effects of tDCS in isolation. Therefore, our data do not address whether modulation of DLPFC activity in itself can induce affective learning biases or alter participants’ mood. However, previous studies have demonstrated that the effects of tDCS tend to be more pronounced when coupled with a task which engages the relevant brain regions (Brunoni et al. 2014; Clarke et al. 2014; Segrave et al. 2014). Furthermore, a systematic review of non-invasive brain stimulation studies targeting the prefrontal cortex indicated that a single session of tDCS does not impact on mood in healthy adults (Remue et al. 2016). It is therefore unlikely that inclusion of a tDCS with sham training condition would have resulted in learning biases or acute changes in mood in this sample. However, we note that a recent meta-analysis reported weak but significant beneficial effects of anodal prefrontal tDCS on stress-related emotional reactivity (Smits et al. 2020). It is thus possible that prefrontal stimulation affected specific aspects of emotional processing which were not measured in the present work.

A further limitation is that the facial expression recognition task (FERT) was not carried out prior to but only after training in order to reduce session duration and thus limit potential fatigue. This design enabled us to compare affective biases after sham versus after active tDCS for both training manipulations, but did not allow for an assessment of any within-session pre-post training changes from baseline that might have been caused by the training itself.

Effects of single cognitive modification training sessions tend to be mild, particularly in healthy participants (Hallion and Ruscio 2011). We can therefore not exclude the possibility that stronger effects of training or tDCS would be observed following multiple sessions. From a therapeutic point of view, it is also worth considering whether future training modifications should include a specific learning criterion in place of a fixed number of trials. This manipulation may enhance specification of the required dosage for behavioural changes and optimise training effects for individuals.

## Conclusion

In summary, the present study provided preliminary evidence that a novel training paradigm could induce a negative cognitive bias in healthy adults. This effect was particularly pronounced in trials in which ambiguous outcomes were used to guide future choices. We found no evidence for an augmenting effect of tDCS over the prefrontal cortex on this form of training. Overall, the findings provide some support for the hypothesis that training with an Information Bias Learning Task can modulate cognitive mechanisms that have been associated with attention and memory biases in depression. An outstanding question, with important implications for depressive symptoms, is whether the training procedure can be adapted to also enhance learning from positive events. Additional research is warranted to investigate the robustness of the effects reported here and assess whether they can be extended to clinical populations.

## Electronic supplementary material

Below is the link to the electronic supplementary material.

Supplementary file1 (DOCX 1420 kb)
