# Supplementary material for: Inducing Affective Learning Biases with Cognitive Training and Prefrontal tDCS: A Proof-of-Concept Study
Source: Cognit Ther Res. 2020 Oct 5;45(5):869–84. doi: 10.1007/s10608-020-10146-9 (PMC8550254; doi:10.1007/s10608-020-10146-9)
Supplement: Supplementary file 1 — Supplementary file1 (DOCX 1420 kb) [file 10608_2020_10146_MOESM1_ESM.docx]

**Inducing affective learning biases with cognitive training and prefrontal tDCS: A proof-of-concept study.**

***Supplementary Information***

**Supplementary Methods and Results**

***Model selection***

AIC and BIC values were calculated for the model used for analyses (**Model 1**) and the following five alternative models (summarised in Table S.1):

**Model 2:** Rather than modelling a single inverse temperature value, it is possible that participants display different stochastic choice behaviour for the win and loss outcomes. This model, which has been applied in previous studies of the IBLT (Pulcu & Browning, 2017), therefore incorporates separate inverse temperature parameters for wins and losses as follows:

$P_{(choice=A\left( i \right))}= \frac{1}{1+\exp^{(-{\beta win*rwin}_{\left( i \right)}-{\beta loss*rloss}_{\left( i \right)}))}}$ (1)

**Model 3:** Instead of learning the independent probabilities of win and loss outcomes, participants might take a model-free approach to the task by learning an overall value of each of the presented shapes (Pulcu & Browning, 2017):

${v^{A}}_{(i+1)}=v^{A}+ a*({out}_{\left( i \right)}- v_{(i)}^{A}$) (2)

in which *v*^A^ represents the value of shape “A”, *α* is a single learning rate for updating the value, and *out*_(_*_i_*_)_ is the outcome of trial *i* (i.e., win – loss for shape “A”, which can be -1, 0, or 1). In the first trial, the value of shape “A” is set at 0. The estimated values of the two presented shapes were transformed into a choice probability by applying a softmax function using a single inverse temperature parameter.

**Model 4:** Similar to Model 1, this model calculates two learning rates and one inverse temperature parameter. This model is slightly simpler, however, in that it omits the “tendency” parameter *t* as described in Equation 4.

**Model 5:** A model using a single learning rate for win and loss outcomes combined, with two inverse temperature values for the two individual outcomes.

**Model 6:** The final model was similar to Model 2, with the main difference being that the values for the win and loss outcomes were centred at zero prior to multiplication with the inverse temperature values:

$P_{(choice=A\left( i \right))}= \frac{1}{1+\exp^{(-\left( {\beta win*(rwin}_{\left( i \right)}- 0.5 \right)-\left( {\beta loss*(rloss}_{\left( i \right)}- 0.5 \right))}}$ (3)

As shown in Fig.S.1, Model 1 provided the best fit to the data across the two studies and was therefore selected for the computational analyses.

**Fig.S.1** Sum of AIC and BIC scores for comparator models per subject from all five task blocks in Study 1 and Study 2. Smaller AIC and BIC scores are indicative of a better model fit. Bar represents mean (SD) of the scores across participants.

***Facial Emotion Recognition Task (FERT)***

*Task description*

During the FERT participants were asked to identify six basic emotions (anger, disgust, fear, happiness, sadness, and surprise) in facial expression stimuli derived from Ekman and Friesen’s (1976) Pictures of Affect Series. All emotions were morphed between 0% intensity (neutral expression) and 100% intensity (full expression) in 10% increments. Each emotion was presented in 40 trials, with four examples of each emotion being shown for every intensity (10-100%). In addition, facial stimuli of neutral expressions (0% intensity) were presented in 10 trials. On every trial, the stimulus was presented for 500 ms, after which the image was replaced by a blank screen. After the stimulus had been presented participants had to indicate which emotion was pictured via a button press. Measures derived from the task included accuracy (number of trials where the emotion was correctly identified), reaction time (in ms), and misclassification (number of trials where the participant assigned an incorrect emotion to the presented stimulus) for every expression.

*Results Study 1*

The Facial Expression Recognition Test (FERT) was completed after IBLT training/tDCS in each session. When contrasting performance on the FERT between the two sessions, it was expected that active tDCS combined with negative IBLT training would improve recognition of negative (i.e. fearful, sad, or angry) facial expressions when compared with sham tDCS. We observed main effects for emotion on accuracy (*F*(5,90) = 10.53, *p* <0.001, η^2^ = 0.248), reaction time (*F*(5,90) = 14.08, *p* <0.001, η^2^ = 0.227), and misclassifications (*F*(5,90) = 7.20, *p* <0.001, η^2^ = 0.211), which suggested that participants were better at recognising positive (i.e., happy) than negative expressions (see Figure S.2.). There were no main effects or interactions for tDCS condition on FERT performance (all *p* >0.05), however, indicating that tDCS combined with IBLT training did not affect recognition of facial expressions.


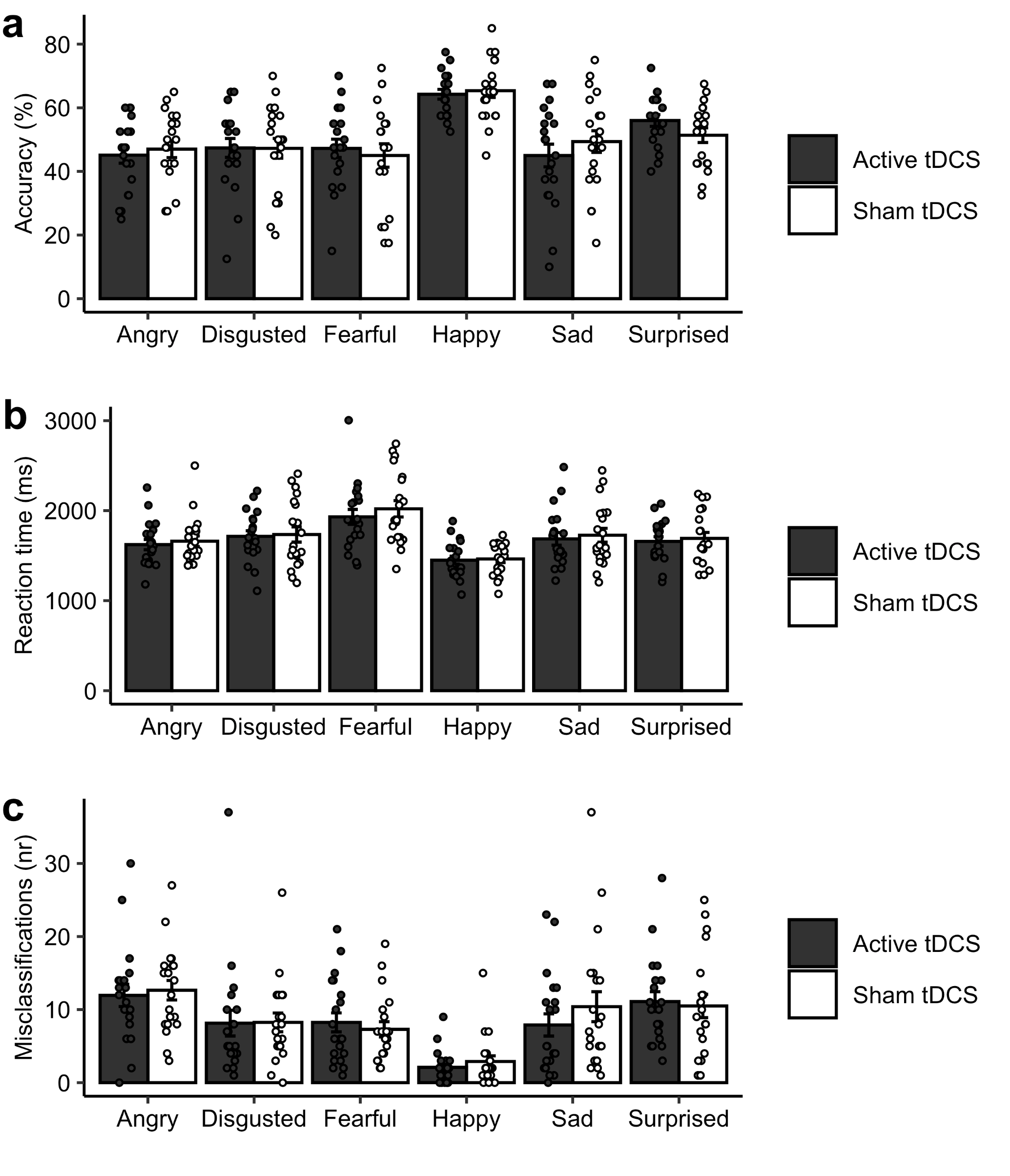


**Fig.S.2** Performance on the Facial Emotion Recognition Test (FERT) following negative IBLT training by tDCS condition. In direct contrasts, participants demonstrated **(a)** higher accuracy (%), **(b)** faster reaction time (ms), and **(c)** fewer misclassifications of happy compared with angry, disgusted, fearful, happy, sad, and surprised expressions. There were no significant effects of IBLT + tDCS on recognition of any of the facial expressions (all *p* >0.05).

*Results Study 2*

In Study 2, it was expected that active tDCS combined with positive IBLT training would improve recognition of positive (i.e. happy) facial expressions when compared with sham stimulation. As for Study 1, main effects were found for emotion on accuracy (*F*(5,90) = 11.41, *p* <0.001, η^2^ = 0.266), reaction time (*F*(5,90) = 18.29, *p* <0.001, η^2^ = 0.169), and misclassifications (*F*(5,90) = 12.64, *p* <0.001, η^2^ = 0.327). On average, participants tended to be more accurate, faster, and less likely to misclassify emotions when presented with happy facial expressions compared to negative emotions. However, there were no main effects or interactions with tDCS condition (all *p* >0.05). There is thus no evidence that active compared to sham tDCS during IBLT training resulted in altered processing of positive or negative facial expressions (see Fig.S.3).


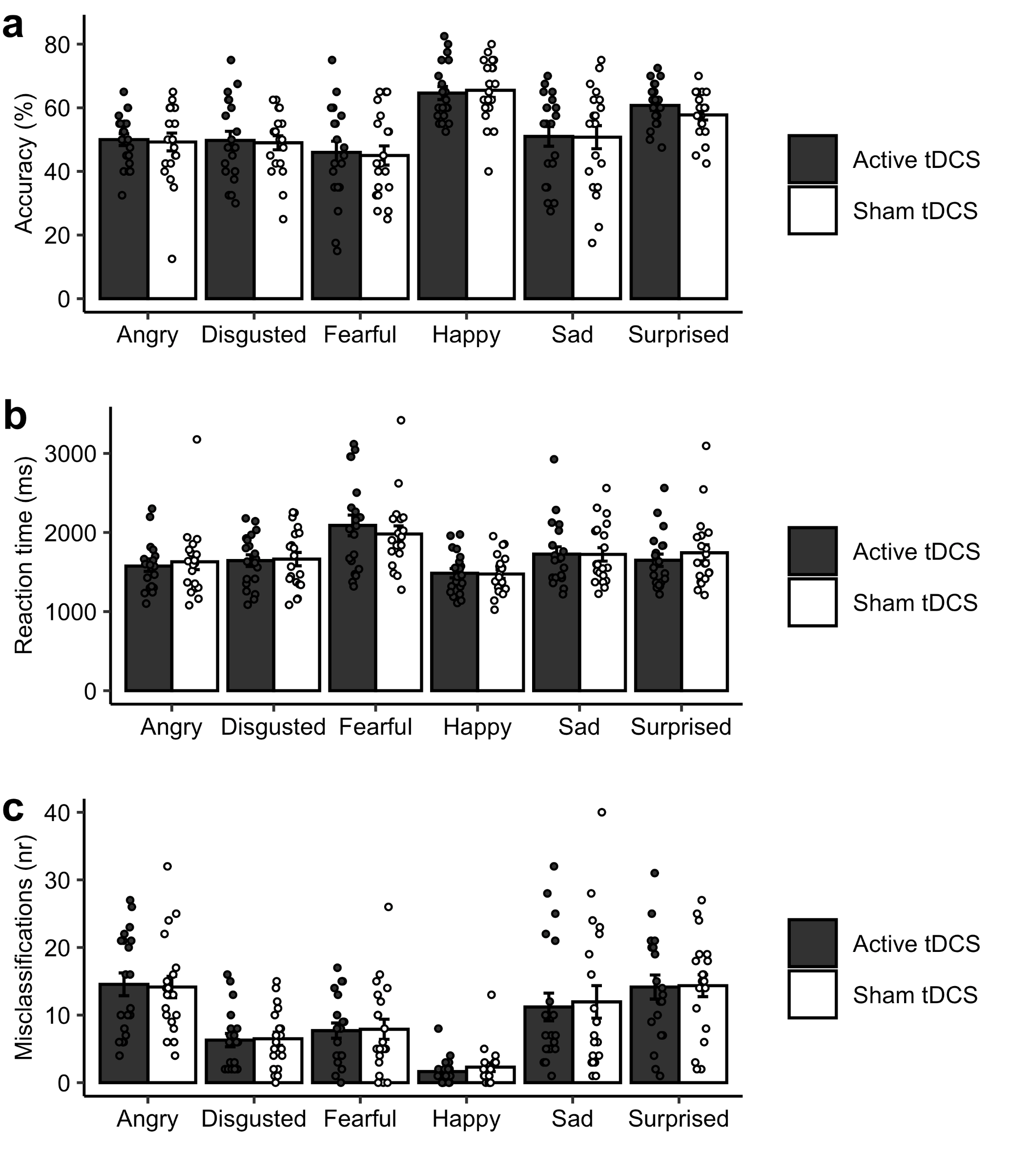


**Fig.S.3** Performance on the Facial Emotion Recognition Test (FERT) following positive IBLT training by tDCS condition. In direct contrasts, participants demonstrated **(a)** higher accuracy (%), **(b)** faster reaction time (ms), and **(c)** fewer misclassifications of happy compared with angry, disgusted, fearful, happy, sad, and surprised expressions. There were no significant effects of tDCS on recognition of any of the facial expressions (all *p* >0.05).

*Comparison of Study 1 and Study 2*To assess the effects of negative versus positive IBLT training on FERT performance, we directly contrasted scores obtained from Study 1 and Study 2. Repeated-measures analyses showed no significant main effects or interactions for either the training type or tDCS condition on emotion recognition (all *p* >0.05). There is thus no evidence that tDCS combined with negative or positive training resulted in changes in positive or negative emotional face recognition.

***Additional results Study 2***

*Computational parameters*

In Study 2, we expected that learning rates for win outcomes would increase over time as participants gained more experience on the task. Contrary to this hypothesis, however, speed of learning for both win and loss outcomes decreased over the course of the ‘Training’ blocks (*F*(1,18) = 16.26, *p* = 0.001, η^2^ = 0.150), with learning rates being higher in Training block 1 than Training block 3 (*t*(79) = 2.46, *p* = 0.016). This may be due to an effect of fatigue, leading to lower learning rates over time.

As for the Study 1, there was a decrease in overall learning rates over time in the ‘Both-volatile’ blocks with IBLT training, with learning rates being higher in the blocks carried out before than after training (*F*(1,18) = 10.88, *p =* 0.004, η^2^ = 0.045). This likely reflects fatigue owing to time on task. However, there was no significant interaction of Outcome valence and Time (*F*(1,18) = 0.43, *p* = 0.519), indicating that the balance between learning rates for wins and losses was unchanged by IBLT training (see Figure S.4.a). Contrary to Study 1, there was thus no trend towards a near-transfer effect of the training. We hypothesised that tDCS would further increase learning from positive outcomes. This hypothesis was not supported. During the ‘Training’ blocks there was no main effect of tDCS (*F*(1,18) = 0.01, *p* = 0.916) and no interaction of tDCS and Outcome valence (*F*(1,18) = 0.026, *p* = 0.873). Similarly, in the ‘Both-volatile’ blocks there were no significant interactions of tDCS with Time (*F*(1,18) = 0.09, *p* = 0.768) or Time and Outcome valence (*F*(1,18) = 0.35, *p* = 0.564). Overall, there was thus no evidence that tDCS of the DLPFC altered learning rates for either positive or negative outcomes in this study (see Figure S.4.b. and S.4.c.). The current protocol aimed to change learning rates without affecting other parameters, such as choice stochasticity. Highlighting the potential of the IBLT training in modulating learning rates in isolation, inverse temperature values remained stable across the blocks and sessions (all *p* >0.05).


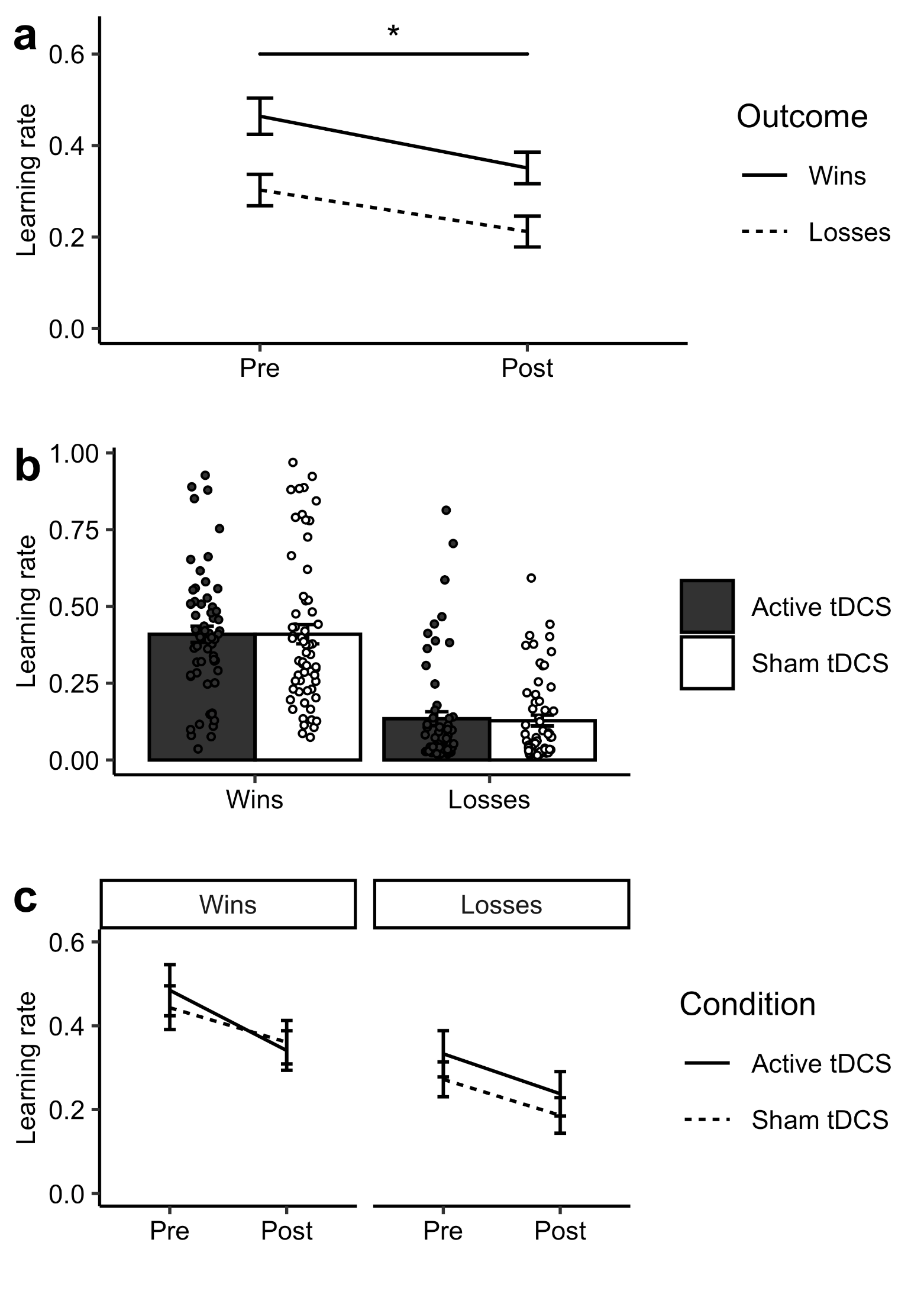


**Fig.S.4** Effects of positive IBLT training and tDCS on learning rates. **(a)** Learning rates for both win and loss outcomes decreased over time in the ‘Both-volatile’ blocks completed before (‘Pre’) and after (‘Post’) training. **(b)** Active tDCS did not alter learning rates for either wins or losses in the ‘Training’ blocks compared to sham tDCS. **(c)** No effect of tDCS was observed for the ‘Both-volatile’ blocks completed before (‘Pre’) and after (‘Post’) positive IBLT training.

*** *p* <.001

*Win- and loss-driven choice behaviour*

We hypothesised that effects of the ‘Training’ blocks would transfer to ‘Both-volatile’ blocks, with the number of win-driven choices being greater after than before training. No support was found for this hypothesis, however, as the difference in win-driven choices between the two ‘Both-volatile’ blocks was not significant (*F*(1,18) = 0.002, *p* = 0.969). Similar to the computational analyses of the learning rates, there thus appeared to be no transfer of learning from positive outcomes from the ‘Training’ blocks (see Fig.S.5.a). Application of active tDCS was expected to increase the number of win-driven choices. Contrary to this prediction, there was no significant effect of tDCS condition in either the ‘Training’ (*F*(1,18) = 0.27, *p* = 0.612) or ‘Both-volatile’ blocks (*F*(1,18) = 1.44, *p* = 0.246). Consistent with the learning rate analyses, this shows that there was no effect of tDCS on affective learning processes as measured with the IBLT (see Fig.S.5.b and S.5.c).


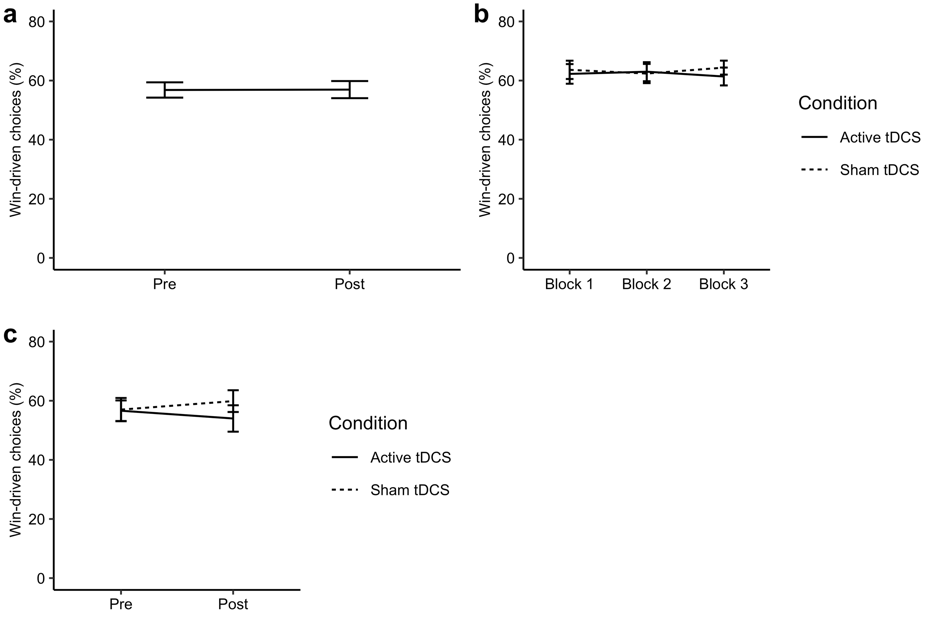


**Fig.S.5** Proportion (*p*) of win-driven choices with positive IBLT training. **(a)** In the ‘Both-volatile’ blocks, there was no significant difference in the proportion of win-driven choices before (‘Pre’) and after (‘Post’) training. There was also no significant effect of active versus sham tDCS in either the **(b)** ‘Training’ blocks or the **(c)** ‘Both-volatile’ blocks. The proportion of loss-driven choices can be calculated as 1-*p*.

*Mood and anxiety measures*We also investigated whether positive IBLT training/tDCS increased resulted in acute improvements in mood and anxiety. Surprisingly, we observed a small increase in scores on the STAI-State over time (*F*(2,38) = 3.40, *p* = 0.044, η^2^ = 0.022), suggesting that subjective anxiety was greater after than before the positive training paradigm. Furthermore, similar to Study 1, a decrease in Positive affect (PANAS) scores was observed over time (*F*(2,38) = 8.00, *p* = 0.001, η^2^ = 0.024). As the training did not near-transfer to the ‘Both-volatile’ blocks or emotional transfer to FERT performance, these changes in questionnaire scores are unlikely to be a direct effect of training. It seems more likely that participants experienced fatigue and reduced interest in the task, resulting in less positive affect over time. There were no changes in PANAS Negative scores over time (*F*(2,38) = 1.43, *p* = 0.252). There was no effect of tDCS on the STAI-S, PANAS Positive, or PANAS Negative scores (all *p* >0.05). Stimulation of the prefrontal cortex therefore did not alter acute mood or anxiety.

**References**

Ekman, P., & Friesen, W. V. (1976). *Pictures of Facial Affect.* Palo Alto, CA: Consulting Psychologists Press.

Pulcu, E., & Browning, M. (2017). Affective bias as a rational response to the statistics of rewards and punishments. *eLife, 6*, e27879.
